# Lectures on the Principles and Practice of Physic; Delivered at King’s College, London

**Published:** 1844-03

**Authors:** 


### REVIEWS.

---

Art. IV.—*Lectures on the Principles and Practice of Physic; delivered at King's College, London.* By Thomas Watson, M.D., Fellow of the Royal College of Physicians, Physician to the Middlesex Hospital, and formerly Fellow of St. John's College, Cambridge. Philadelphia: Lea & Blanchard. 1vol. 8vo. pp. 920. 1844.

These celebrated lectures were delivered at King's College, London, during the medical session of 1836 and '37, and were repeated, with slight variations, for four successive years. To fulfil a rash promise, we are told by the author, it was that they were printed, at length, in the Medical Gazette. They were prepared, it will be seen, especially for students of Medicine, and they constitute, we believe it is conceded on all hands, one of the most complete systems of medical practice to be found in our language. A reprint of these lectures is now offered by Messrs. Lea & Blanchard to the profession of the United States, in a large and beautifully executed volume. It is not our purpose to give a synopsis of the subjects discussed in it, much less to attempt an analytical review of a work embracing the whole range of human maladies, their causes, symptoms and treatment. Ninety lectures, fully written, comprising all that the author has derived from his acquaintance with the best authorities in the profession and his own extensive practice, are not to be compressed, by any process known to us, into the limits at our disposal. Nor would our readers desire such a review. They will prefer studying the subjects at the full length in which they are presented in the ponderous volume. To such however, as may not have the means of possessing themselves of the work immediately, we have supposed extracts from some portions of it might not be unacceptable. In making selections for quotation we have been governed by our own taste, and are far from being sure that we have made choice of the most valuable.

Our first extract relates to *strumous ophthalmia;* a malady especially prevalent among the colored population of our country. Our brethern will welcome any new light that may be reflected on its treatment.

"*Treatment of Strumous Ophthalmia.*—You will readily believe, from what has been said of this complaint, that it is an obstinate and troublesome one. Even when removed, it is very apt to recur again. The scrofulous *habit* on which it depends we cannot get rid of; and whenever the exciting causes of scrofulous *diseases* come into action, this form of scrofula is very prone to declare itself, at the period of life which I have already mentioned.

"More good is to be done by general treatment, applied to the system at large, in this form of ophthalmia, than in those we were occupied with before; and this is one strong point of difference between them.

"In the first place we must endeavor to correct that unnatural condition of the whole system, and especially of the digestive organs, which is commonly so striking a concomitant of the local disease. It will be proper to clear out the bowels in the outset, and occasionally, by a mercurial purge; and to regulate them at other times by laxatives, such as rhubarb, or the confectio sennæ, or castor oil. The recovery will be greatly promoted also by those measures which are found to benefit the general health in such constitutions; warm clothing, frequent ablution of the body, nourishing though plain food, the respiration of a pure atmosphere, *change* of air, and regular exercise.

"In addition to these measures, tonic medicines should be administered; the preparations of iron, for example, or the dilute mineral acids; but the best remedy of this kind is, undoubtedly, the sulphate of quina. This may be given to a child in grain doses, three times a day, dissolved in water, with a drop of the dilute sulphuric acid, and some syrup of orange peel. Dr. Mackenzie, in particular, has put this medicine fairly to the test, having employed it in a very large number of cases with the happiest results. In most of his patients he declares that it acted *like a charm*, 'abating, commonly in a few days, the excessive intolerance of light and profuse epiphora; promoting the absorption of phlyctenulæ, and hastening the cicatrization of ulcers of the cornea.' And Mr. Lawrence adds his testimony to the same effect; and his experience in this disease, like Dr. Mackenzie's, has been large enough to make it highly valuable.

"A few words will suffice to explain the kind of local treatment that has been found useful. You may feel tempted to apply leeches round the eye. This is seldom requisite, except when there is more redness and pain than common, and the tongue becomes white, and the skin hot. Certainly you must not take the intolerance of light as a fit indication for the use of leeches. Abstraction of blood rather aggravates that symptom, apparently by increasing the irritability of the retina. Warm fomentations are generally very comfortable to the patient's feelings.

"When the general disorder of the system has been somewhat rectified, local stimulants and astringents are of great service. The vinum opii, and the solution of lunar caustic, are the best. These are often tedious cases, and therefore it is necessary that you should be aware of one great objection to the long continued employment of the nitrate of silver wash, which objection has been pointed out by Dr. Mackenzie. It is apt (but only when frequently repeated for a long time together) to stain the conjunctiva of an indelible olive color. For this reason the vinum opii is to be preferred in slow cases, and in cases where frequent relapses happen. The good effects of either of those preparations are very striking; they diminish the irritability of the eye, and promote the healing of the ulcers. The red precipitate ointment, and the citrine ointment of the Pharmacopœia diluted, are also found beneficial.

"Counter-irritation is another local measure, which is of undoubted utility in this complaint. A great change for the better in the state of the organ often occurs, almost suddenly, upon the rising of a blister placed behind the ear, or at the back of the neck. And issues in the arms are not only serviceable in promoting the cure, but have a marked effect in many children in preventing relapses. Mr. Welbank, in his notes to Frick's *Treatise on Diseases of the Eye*, says that he has seen chronic strumous ophthalmia, of seven years' duration, quickly and effectually relieved by an issue in the arm. Having once (says he) in the case of a boy in Christ's Hospital, directed the healing of an issue which had been made above twelve months, I found the immediate consequence to be a relapse of strumous inflammation and ulceration of the cornea, resisting every measure but the renewal of the issue.'

He suggests also (what parents are sometimes more willing to assent to) the advantage of making counter-irritation by piercing the lobe of the ear, and inserting a ring or silk; and 'a very convenient form of vesication will be found in the application of a strong thread, smeared with *emplastrum cantharidis*, and firmly tied behind the ear at the angle of its reflection.'

"When ulceration is going on in the cornea, and threatening to penetrate it, the progress of the ulcer may be checked by touching the surface once in two or three days with a pencil of lunar caustic which has been scraped to a fine point.

"When the more urgent symptoms have abated, and the discharge of hot and irritating tears has ceased, the crusta lactea may very easily be got rid of. The crusts are to be removed by a light poultice, or by warm water; and then the part must be bathed from time to time with a lotion made by mixing the oxide of zinc with water; a drachm to four ounces is the proportion I am in the habit of prescribing. If rose water be used instead of common pump water, the prescription will be thought the more *elegant.* This lotion will speedily dry up the discharge, and in a short time no trace of the ugly looking crust will remain. Parents are highly delighted and very thankful when you thus accomplish the removal of a large disfiguring and disgusting scab, which they naturally enough felt apprehensive might leave behind it a corresponding scar. But it is quite superficial."

We make no apology for introducing so long an extract, since we do not believe that we could fill our pages with matter of more decided practical value. The following quotation is not so long—it contains a suggestion never to be overlooked in cases of palsy. Our author remarks—

"There is one very common and distressing consequence of such disease of the spinal marrow as produces paraplegia, not particularly noted in any of the cases which I have related, but always to be looked for. The muscles, by means of which the bladder empties itself, are apt to participate in the palsy; and then the bladder empties itself no longer. The urine accumulates in it, and distends it, and the ureter even becomes distended; and in this way not only the present but the prospective danger is increased. For the foundation of future disease in the kidneys is often thus laid, even when such distension of the bladder by its retained contents occurs independently of any disease of the spine; as it may do from stricture; from enlargement of the prostate; or even from the voluntary retention of the urine beyond a certain period, through feelings of delicacy. You are to look out, I say, for this distention of the bladder, and relieve or prevent it by the introduction of a catheter through the urethra. You must not be deceived by being told that the patient passes plenty of water; that it even runs from him. Incontinence of urine is, in fact, in these cases, though it may sound paradoxical, a sign of retention of urine. The urine dribbles away because the bladder admits of no further distension; it overflows, and runs out at the natural orifice, but the bladder remains constantly full and stretched. You must make an examination, therefore, of the hypogastric region with your hand. If you find that part of the belly hard and resisting, and giving out a dull sound on percussion, you may be sure, in these cases (where there is paralysis of the lower extemities, and the water dribbles away), that the bladder is full, and has lost the power of expelling its contents. Sometimes you may recognize the fluctuation of the urine in the distended bladder, and ascertain the globular shape of that organ. It will rise even beyond the umbilicus. But what I chiefly wished to point out to you is, the circumstance that the bladder becomes diseased, and the urine altered in quality, under this state of palsy. The urine becomes thick, ropy, and alkaline, and exhales a very offensive ammoniacal smell; and the inner surface of the bladder is found, after death, to be thickened, red, and covered with adhesive mucus—in a state of chronic inflammation, in short.

The facts related in the following passage, taken from the lecture on *hydrophobia* will be read with interest:

"It is curious that different species of animals appear to be susceptible of hydrophobia in different degrees. Thus, according to Mr. Youatt, two dogs out of three, bitten by one that is rabid, become rabid. The majority of horses inoculated with the virus perish. Cattle have a better chance: perhaps because in them the skin is looser and less easily penetrated. A full half (he thinks) of those that were seized by a mad dog would escape. With sheep the bite is still less dangerous.

He reckons that not more than one in three would be affected. The tooth, perhaps, has been wiped clean in its passage through the wool. The human being is least of all in danger. John Hunter states that he knew an instance in which, of twenty-one persons bitten, one alone became affected with hydrophobia. Dr. Hamilton estimates the proportion to be one in twenty-five. But I fear these computations are much too low. In 1780, a mad dog, in the neighborhood of Senlis, took his course within a small circle, and bit fifteen persons before he was killed: three of these died of hydrophobia. The slaver of a rabid wolf would seem to be highly virulent and effective. These beasts fly always, I believe, at a naked part. Hence, probably, the fatality of their bites. The following statement applies exclusively to the wolf. In December, 1774, twenty persons were bitten in the neighborhood of Troyes; nine of them died. Of seventeen persons similarly bitten in 1784, near Brive, ten died rabid. In May, 1817, twenty-three persons were bitten, of whom twelve died of hydrophobia within two months. Hence we have one hundred and fourteen persons bitten by rabid wolves, and among them no less than sixty-seven victims; considerably more than one half. There is no doubt, however, that a majority of persons who are bitten by a mad dog escape the disease. This may partly be owing to an inherent inaptitude for accepting it. We see some persons who, though often in the way of it, do not contract syphilis; there are others upon whom the contagion of small-pox has no influence. This difference exists, apparently, even among dogs. There was one dog, at Charenton, that did not become rabid after being bitten by a rabid dog; and it was so managed, that, at different times, he was bitten by thirty different mad dogs; but he outlived it all. Much will depend also upon the circumstances and manner in which the bite is inflicted; if it be made through the clothes, and especially through thick woollen garments, or through leather, the saliva may be wiped clean away from the tooth before it reaches the flesh. In the fifth volume of the Edinburgh Medical and Surgical Journal, there is a case described by Mr. Oldknow, of Nottingham, in which a man was bitten in three different places by the same dog; viz: in the scrotum, the thigh, and the left hand; the bite on the hand was the last. Now it seems not improbable that but for this last bite, on a naked part, he might have escaped. At least it was a remarkable circumstance that the phenomena of recrudescence occurred only in the hand and arm. The dog is supposed to have closed his mouth after inflicting the first two bites; and thus to have charged his teeth afresh with the poisonous saliva.

"It is this frequent immunity from the disease in persons who have been bitten, that has tended to confer reputation upon so many vaunted methods of prevention. Ignorant persons, and knavish persons, have not failed to take advantage of this. They announce that they are in possession of some secret remedy which will prevent the virus from operating. They persuade the friends of those who die that the remedy was not rightly employed, or not resorted to sufficiently early; and they persuade those who escape that they escape by virtue of the preventive remedy. If the plunder they reap from the foolish and the frightened was all, it would be of less consequence; but unfortunately the hope of security without undergoing a painful operation leads many to neglect the only sure mode of obtaining safety."

Dr. Watson entertains the opinion common among English writers on the complaints of females, that puerpural fever is a contagious disease, and hence recommends the following precautions:

"I need scarcely point to the practical lesson which these facts inculcate. Whenever puerperal fever is rife, or when a practitioner has attended any one instance of it, he should use most diligent ablution; he should even wash his hands with some disinfecting fluid, a weak solution of chlorine for instance: he should avoid going in the same dress to any other of his midwifery patients: in short, he should take all those precautions which, when the danger is understood, common sense will suggest, against his clothes or his body becoming a vehicle of contagion and death between one patient and another. And this is a duty so solemn and binding, that I have thought it right to bring it distinctly before you."

Our next quotation relates to a disease, the first description of which was published but a few years ago. We shall devote much space to an account of this affection, assured that Dr. Watson's remarks concerning it convey information which many will be glad to receive:

"*Bright's Disease.*—Now it is quite certain that the presence of albumen in the urine does often acccompany and bespeak a very serious organic disease of the kidney. For this disease we have no appropriate name. I wish we had. Some call it

*granular degeneration* of the kidney, but the epithet granular is not always applicable. It is most familiarly known, both here and abroad, as *Bright's kidney*, or *Bright's disease;* after the eminent physician, who, in 1837, first described it, and showed its great pathological importance. These are odd-sounding and awkward terms; but in the lack of better, I must employ them.

"*Anatomical Characters.*—It is very difficult to describe, in words, the anatomical characters proper to this renal disease; for they are neither very definite nor very constant. The description that I am about to attempt will be made more intelligible by Dr. Bright's plates, and those of M. Rayer, which are both before you.

"The morbid appearances presented by the kidney are such as denote some change in its intimate structure. Its cortical (or secreting) portion is the primary and chief seat of this degeneration; yet what is called its medullary (*i. e.* its excreting) part, is also sometimes implicated, but in a less degree.

"These morbid appearances relate to the size, figure, and consistence of the kidney; to the color and condition of its surface, and of its interior. With respect to some of these points there is much variety in different cases; and studying this variety under the light which is thrown upon it by the clinical history of the disease, we have reason to believe that it is connected with different stages of the disorganizing process. Thus if we look to the *size* of the diseased organs, they are sometimes much larger than natural, sometimes of the ordinary magnitude, sometimes considerably smaller. The average weight of the adult human kidney is four ounces. M. Rayer has met with some, in this disease, weighing twelve ounces. Both the increment and the decrement of the natural bulk belong principally, if not altogether, to the outer secreting portion of the gland. If a longitudinal section of the exaggerated kidney be made, its cortical part is seen to be unduly broad; and the same part is disproportionally narrow when the whole organ is smaller than common. For this reason, in the latter case, the radiating medullary portions approach nearer to the surface than they are observed to do in a healthy kidney. And it furthermore appears that the enlargement is most commonly coincident with the earlier, and the contraction or shrinking with the later, stages of the renal disease.

The *consistence* of the diseased gland is variable also. Sometimes, and for the most part in the earlier periods, it is soft and flabby; sometimes, and especially in the later periods, it is remarkably compact and hard. The size and consistence of the kidney are, in most cases, inversely proportioned to each other.

"Again, the *form* of the kidney, in the disease in question, often undergoes some modification. As the special change proceeds, the exterior of the gland shows a tendency to become indented by linear depressions, and to present a lobular shape. This, however, is by no means a constant phenomenon, even in the most advanced stages of the malady.

"When its proper investing tunic is stripped off—and less distinctly *through* the same tunic, before its separation—the surface of the kidney appears mottled, marbled, or stained, of a yellowish gray color in one place, and of a dark or purple tint in another. Occasionally it is pale throughout its whole extent; more commonly of divers hues, and variegated with little streaks, which are portions of vessels containing red blood. Sometimes the surface is curiously speckled; often uneven as if strewed with prominent grains; in some instances quite rough and scabrous. These several unnatural appearances are usually the more conspicuous, in proportion as the complaint is the more advanced.

"The most uniform, however, and the most characteristic of the morbid appearances, are those presented by the cut surface of the kidney, when it has been divided into two symmetrical portions by a longitudinal incision. We then perceive that the cortical substance is the main seat of the morbid alteration. It has lost, in a greater or less degree, its proper red color and uniform aspect. Sometimes it puts on a speckled or granular appearance; but this, in my experience, is less common than a pale, homogeneous surface, somewhat like the section of a parsnip. Its natural striæ are confused or obliterated. The incised surface gives one the notion of some deposite, whereby the original texture of the part is obscured. The blood-vessels seem, many or most of them, to have been emptied by compression, or to be blocked up by yellowish solid matters; while the healthier pyramidal masses belonging to the medullary portion of the kidney are displaced, and pushed aside, or encroached upon by the same yellowish matter, which sometimes interposes itself between, and opens out, their radiating tubuli. Together with these changes of appearance and structure, I have several times found the veins that emerge from the kidney firmly plugged up by coagula of blood.

"In some rare cases the kidney is studded, both on its surface and throughout its interior, with numerous small cysts or cells, containing a thin transparent liquid. These cysts have been inaccurately termed hydatids. It is not at all uncommon to meet with one or two such cysts in this diseased state of the organ.

"It has been made a question whether the various appearances which I have been attempting to describe, and which sensibly differ in degree and combination in different cases, are characteristic of different morbid conditions, or merely of different stages and varieties of the same essential change. Our knowledge of the subject is scarcely sufficient to supply a positive solution of this question. Excepting perhaps the cysts, my own *opinion* is, that they are all accidental forms and effects of one and the same morbid process. At the same time I ought to tell you that both Dr. Bright and Dr. Christison appear to incline to the opposite conclusion.

"There is still another state of the kidney, very different to the eye from any that I have yet mentioned, but which has been thought, and which I think, to be, in some cases at least, the first stage of all in the disorganizing process. This state, which I referred to when speaking of suppression of urine, may be briefly described in two words—*sanguine congestion.* The whole organ is gorged with blood, which sometimes drips freely from it when it is cut open. The kidney is in general large, somewhat flabby, of a deep dark red, even of a chocolate or purplish color, nearly uniformly diffused, except that the cut surface is usually diversified by still darker tuft-like spots, which have been ascertained to be the Malpighian bodies filled with blood. This change from the natural appearance of the kidney is evidently of a recent kind; and the symptoms that have been observed to belong to it are these: Fever, preceded often by rigors; uneasiness or dull pain in the loins; nausea and vomiting; a very scanty secretion of urine, which is sometimes tinged with blood, and always albuminous; occasionally complete suppression of urine. To these symptoms there is presently added, in most cases, sudden and general anasarca—what is commonly called inflammatory, active, or febrile dropsy. If the secretion of urine be entirely suspended, death soon ensues by coma, as I explained to you yesterday; but if not, the disorder is frequently fatal by the supervention of some acute internal inflammation; pleurisy, or pericarditis, or pneumonia, or peritonitis. Many persons recover completely from the condition expressed by this combination of phenomena. Many seem to recover, but bear about with them the germs or beginnings of those more chronic and latent changes which constitute 'Bright's kidney.'

"*Symptoms.*—And what are the signs which indicate, to an instructed eye, the presence of those changes? Some of them are precisely the same, in kind, as those which denote the acuter disorder; only mitigated in degree, and of slower march and succession. The patients are subject to obscure lumbar pains; to sickness from time to time, and retching; and their urine is apt to be red, brown, or dingy, as well as albuminous, from the intermixture of some of the coloring matter of the blood. They are obnoxious to inflammations of the serous membranes also; and more particularly to head affections, of which they often die; drowsiness, convulsions, apoplexy. And, to finish the resemblance, many of them, ay, most of them, become at length anasarcous. Besides these symptoms, there are others which are not seen in the acute malady; but it is acute. Gradually increasing pallor is almost constant; disease of the heart is common; and the skin, in general, even in the absence of fever, is remarkably dry and unperspiring. The patients are troubled by a frequent want to make water; by flatulence of the stomach and intestines; and by caprice of the bowels, which are sometimes obstinately costive, sometimes prone to diarrhœa.

"Now it is worth your while to remark, with respect to this category of symptoms, that (the state of the urine excepted) they have no special *primâ facie* reference to renal disease. They are all common enough in various other complaints. In truth they are mere secondary consequences of Bright's disease; and in so far as they are symptoms of it, they are *indirect* symptoms. Before Dr. Bright no one perceived, in such symptoms, any indications of disease of the kidney. The primary and fundamental organic malady reveals itself by no indirect symptoms, excepting those which are furnished by the urine.

"*Albuminous Urine.*—Seeing, then, that this peculiar disease of the kidney is coupled with effects so grave and perilous, and seeing that one of its most positive and distinctive marks is an albuminous state of the urine, two questions of great interest at once present themselves.

"1. Does albuminous urine *always* imply the presence of Bright's disease?

"2. Is Bright's disease, when present, *always* accompanied by albuminous urine?

"To both these questions the answer is—no.

"It is certain that some articles of food have the effect, in some persons, of rendering the urine for a time albuminous: perhaps it would be more correct to say that certain forms of indigestion cause this change. Albumen has also been detected in the urine under that general state of irritation produced occasionally by mercury, or by a blister to the skin. In the crisis of some febrile disorders, and in some cases of pregnancy, the same phenomenon has been observed. Whenever blood, proceeding from any part of the long tract of mucous membrane which lines the urinary organs, mingles with the urine, that fluid of necessity contains albumen, and coagulates if tested by heat or by nitric acid.

"On the other hand, when the kidney is really affected in the way already described, the admixture of albumen with the urine is apt to disappear, for a while, even suddenly. I have known it to vanish for several hours, immediately after the effectual application of a hot air bath; and after profuse purging by a full dose of elaterium. Sometimes it is absent for a longer period.

"Another important question, therefore, now arises. Finding albumen in the urine, how are we to know whether it does or does not indicate the presence of Bright's kidney?

"We may judge, in part, by frequently testing the urine, and noticing whether the albuminous impregnation be transitory or persistent. In part also we judge by the absolute amount of the albumen in a given measure of urine. If the urine be deeply charged with that unnatural ingredient, the presumption is strong that the kidney disease is in progress, and, when that disease is confirmed, another remarkable change is found to have taken place in the urine. Its specific gravity is very low; and striking in contrast with that of diabetic urine. This is therefore a very strong additional diagnostic circumstance.

"On Dr. Prout's authority we have assumed the specific gravity of healthy urine to range between 1015 and 1025. Other writers make it higher. But the urine voided in Bright's disease is sometimes as low as 1004; and its mean specific gravity does not exceed 1013.

"I need scarcely again remind you, that the question of specific gravity must always be viewed in relation to the absolute quantity of urine secreted. The specific gravity depends, of course, upon the proportion of the solid constituents of the urine contained in a given quantity. If the aqueous portion be augmented, the effect upon the absolute density will be the same as if the solid contents were proportionally diminished. But when, as frequently happens in certain stages of this renal disease, the specific gravity decreases while the quantity of the urine decreases also, that conjunction of phenomena becomes especially significant.

"The density of the urine being thus unnaturally low, notwithstanding the addition of the new substance, albumen, it follows, as a matter of inference, that the solid constituents proper to healthy urine must be sensibly diminished; and they are found, in fact, to be so. These solid ingredients consist mainly of urea, and of certain salts. The aggregate solid contents amount, in health, to sixty-seven or sixty-eight parts in every 1000. In Bright's disease the amount has been ascertained to be diminished to twelve or fourteen parts, and even, in an extreme case, to less than this—to about six parts.

"The urine contains, then, albumen; and it is deficient in urea. These two facts suggested, naturally enough, to M. Solon, and to others, the notion that albumen might be formed by a sort of conversion, at the expense of the urea; since these substances, by a slight alteration in the ratio of their elements, pass respectively each into the other. But it is not so. Dr. Christison had observed many years ago, that when the urine was deprived of the greater part of its urea, the quantity of albumen contained in it was small; and, on the other hand, in cases where the urea was considerable in quantity, the albumen was plentiful also. In a recent work on this subject, the same physician states that the whole of his subsequent experience has been in conformity with this observation.

"It being certain, therefore, that the albumen is not vicarious of the urea, what (you may ask) becomes of the urea? It is detained in the blood; and may readily be recognized there in considerable quantity; and herein lies, as I conceive, the secret of the secondary affections which belong to this disorder, and of its great fatality. The body is poisoned in detail by the retention of its own excrements. The blood not being duly purified through that great emunctory, the kidneys, is spoiled for its purpose of nutrition. Besides containing urea, it undergoes other and more manifest changes. Its proportion of fibrine varies; and it gradually becomes poor in coloring matter; the serum is less albuminous also, and of a lower specific gravity, than in health. The quantity of albumen in healthy blood averages from sixty-five to sixty-nine parts in 1000. In this malady Dr. Babington has found it reduced to sixteen parts. The average specific gravity of healthy serum is 1030; but in Bright's disease it descends to 1024, 1020, and even to 1013. Now Dr. Christison has made out the very interesting fact, that there is a definite inverse ratio between the coagulability of the urine, and the density of the serum. The more albumen there is in the former of these fluids, the less is there in the latter, and the lower is its specific gravity. So that the deficiencies of the one fluid balance the superfluities of the other. All this is very different from what takes place in diabetes, in which sugar is excreted with urine that is otherwise healthy; whereas, in Bright's disease, urea, which ought to be discharged, remains in the blood; and albumen, which ought not to be separated, is taken from the blood, and carried out with the urine.

"*Stages.*—I have now described the changes presented by the kidneys in this disorder, the symptoms which attend it, and the morbid conditions both of the urine and of the blood. But these all vary and fluctuate at different periods of the complaint. I must next, therefore, endeavor to state what has been ascertained by its course and progress.

"When the chronic disorder is not a legacy left by the more severe and acute form of disease which I have termed febrile dropsy, it is apt to creep on very insidiously, and to escape our notice—and its history is not fully known. It will be enough if I distinguish two stages of the malady—the early, and the advanced.

"In the early stage the urine is generally scanty. Instead of about 40 ounces in the twenty-four hours, the patient voids 16, 12, 8, or even as little as 2 or 3 ounces. Sometimes the secretion is nearly or quite suppressed; and then the head seldom fails to become affected in the way already described. The urine has also an unnatural appearance. It is red, or dark, obscurely turbid, like muddy beer. It froths more than usual; and if you blow into it through a tube, you raise bubbles similar to those which may be formed in soapy water. Its specific gravity is somewhat, yet not greatly reduced; about 1021, perhaps; it is seldom at this period as low as 1016. It contains an abundance of albumen.

"At the same early period, blood drawn from the arm exhibits the buffy coat. The serum is much diminished in density, and contains a considerable quantity of urea. There is no decrease in the fibrine; perhaps it is a little augmented; and there is no great change in the amount of coloring matter.

"In the more advanced stages of the disease, the quantity of urine is frequently not below the standard of health; and it sometimes considerably exceeds that standard, so as to constitute one variety of chronic diuresis (*anazoturia*), which some call diabetes insipidus. It is usually pale, slightly opaque, and of a very low specific gravity; 1014, 1010, 1007. Once, when the quantity of the urine was *not* in excess, Dr. Christison found the specific gravity to be no more than 1004. There is a corresponding reduction in the natural solid ingredients of the urine. Albumen, too, is present, but more uncertainly than in the early periods: fluctuations in this respect are more common than before. It is a mistake to suppose that the amount of albumen increases as the disorder advances. The contrary rule would be more near the truth. In general the albumen is plentiful and almost constant in the outset of the malady; less surely present as it proceeds; and sometimes entirely absent in its latter periods; and it is of importance to remark that the alteration in the specific gravity follows the opposite law. The declension of density, so far from being corrected, augments with the progress of the disorder. Hence the one of these morbid phenomena is a valuable check upon the other, considered as an index of what is going on in the kidney.

"And another fact, which it is essential for you to know and to remember, is, that in any stage of the disease, the supervention of febrile disturbance, from local inflammation or whatever other cause, tends to renew, for the time, those qualities of the urine which belong to the early period.

"Meanwhile, the disease advancing, the serum of the blood recovers more or less its lost specific gravity, in proportion to the decrease of albumen in the urine. The quantity of fibrine seems, in some cases, to diminish. But the striking and most characteristic change is the rapid disappearance of the coloring matter, the hematosin, as it is called. This may at length be so much reduced as to form less than a third of the healthy average. If venesection be occasionally employed, this process of depravation is accelerated; but it takes place whether blood be artificially withdrawn from the system or not. 'I am acquainted,' says Dr. Christison, 'with no natural disease, at least of a chronic nature, which so closely approaches hæmorrhage in its power of impoverishing the red particles of the blood.' Hence the peculiar pallid or dingy hue of the patient's skin; the leucophlegmatic and even waxy aspect which invariably stamps the victim of this complaint.

"These characters, then, of the urine and of the blood, when rightly compared and interpreted, reveal not only the existence of the renal disease, but also, with much probability, the stage or degree that it has reached.

"*Secondary Affections.*—Let us next review, a little more in detail, those secondary affections which I have already pointed out as being incidental to the subjects of this renal malady. They are of much consequence; for, in the course of the disease, more or fewer of them are almost sure to occur; most of them are productive of very serious distress; and some of them place the patient's life in immediate jeopardy, and often bring it to a premature end. Moreover it is by these secondary affections that our suspicion of the primary disease upon which they depend is, in general, first awakened; and it is to the prevention or the removal of these same secondary affections that our curative endeavors must chiefly be directed.

"The most common, and practically the most important, of them all, is anasarca; but of this, although I mention it first, I shall postpone, for a while, the further consideration.

"Another very common, and very important secondary complication, is the occurrence of what we compendiously call *head symptoms:* various manifestations of derangement in the cerebral functions; headache, drowsiness, delirium, epileptic seizures, apoplexy. So frequently, indeed, is the death of the patient preceded by coma, with or without convulsions, that Dr. Christison considers this to be the 'natural termination' of the disease, or 'the mode in which it proves fatal when life is not cut short by some other incidental or secondary affection.' Of seventy fatal cases observed by Dr. Bright, death was ushered in by well-marked cerebral symptoms in thirty.

"I have already told you the circumstances under which these affections of the brain usually arise. They almost always follow any great diminution, or the entire suspension, of the secretion of urine. But this rule is not so strict as to admit of no exception. Occasionally, but I believe very seldom, the urine, in this disorder, is reduced to a very small amount, while the head remains undisturbed. Of this Dr. Christison has recorded a remarkable instance. One of his patients voided no more than two ounces of light urine daily, for nine days before his death; yet he continued sensible to the very last minute of his existence, and died simply of inanition. Sometimes apoplectic symptoms occur, and carry the patient off, although there has been no extreme or material reduction in the quantity of urine.

"Now when death has thus taken place in the way of coma, and the case had been complicated with anasarca, and serous liquid is found accumulated in unnatural measure in the cerebral ventricles, and in the tissue of the pia mater, it seems reasonable to ascribe the coma to the presence and pressure of that liquid. The dropsy has extended to the brain. And this view of the matter is strengthened by the connection which may sometimes be noticed between the accession of coma and the visible increase of the dropsy in other parts of the body. My own experience accords entirely with that of Dr. Christison, as expressed in the following statement. 'If the dropsical fluid be allowed greatly to accumulate, drowsiness, the first symptom of the affection of the head, very soon makes its appearance in the generality of cases, and it will speedily pass to fatal coma, if not controlled, but the removal of the dropsy will usually remove the drowsiness.'

"To many cases, however, this explanation will not apply, there being no morbid collection of water within the skull, nor any other appreciable change there; nor, perhaps, any dropsy elsewhere. In such cases we refer the ultimate symptoms, the stupor and the death, to the poisonous influence of the urea in the unpurified blood upon the organs of animal life. Yet this explanation also has its difficulties. Urea must often circulate with the blood without affecting the brain. Dr. Christison states that he has repeatedly known the daily discharge of the solids of the urine to be reduced, for weeks together, to one-fourth of the natural amount, while, moreover, analysis of the blood showed that it was loaded with urea, without the appearance of any head symptom. Dr. Bright also relates a case to the same purpose. A person laboring under this disease of the kidney lived for four or five years under his occasional observation. The blood was analyzed in the earlier stage, and found to contain a large quantity of urea; as much as the urine itself contained. Yet this patient had no *fits* till towards the close of his life.

"I have sometimes fancied that the pale and watery condition to which the blood is at last reduced, may have something to do with the stupor and coma. I showed, you, some time ago, when speaking of spurious hydrocephalus, that similar symptoms are apt to ensue, in conjunction with a similar defect of hematosin. It would seem that, under such circumstances, the functions of the brain are exercised irregularly, languidly, and at length not at all, in consequence of the failing supply of its appropriate stimulus through the arteries.

"Another striking circumstance, observable in this disease, is a readiness of various organs of the body to inflame, and particularly of the serous membranes. According to M. Solon, who has lately published a thick volume on *Albuminurie*, this disposition has not been so manifest in France; but of its frequent appearauce in this country I can add my own testimony to that of Dr. Bright, of Dr. Christison, and of Dr. Gregory. Such intercurrent acute inflammation is not an uncommon cause of the patient's death. The pleura appears to be much more often affected in this manner than either the peritoneum or the pericardium.

"It follows from this tendency, that when we come to inspect the dead body, we seldom find the kidney to be the only part in which structural changes are plainly visible. Most commonly evident traces of disease are met with in various organs.

"Disorder of the stomach and bowels is certainly a frequent companion of the renal malady: nausea, vomiting, flatulent distension, diarrhœa.

"It would appear, however, that these incidental and secondary complications prevail with irregular frequency in different places. They are probably determined, in some measure, by local and peculiar agencies. Thus vomiting and diarrhœa have been more familiar to the Edinburg observers than in London to Dr. Bright, or in Paris to M. Solon: while the headaches and coma, so often witnessed by the British physicians, have been comparatively uncommon in France.

"Disease of the heart, if not a secondary consequence, is a very frequent accompaniment of Bright's kidney. It is probable that the cardiac disease, and the renal disease, have sometimes no connection in respect to cause and effect, but are both results of some common cause; of habitual intemperance, for example.

"I am, however, of opinion, that the renal malady has a direct tendency, by its effect upon the blood, to generate disease of the heart. It induces anæmia; and anæmia, as I showed you on a former occasion, implies debility of the muscular texture of the heart, and leads to dilatation of its cavities; and the weak muscle, becoming irritable also, grows thicker as it labors more. In fact, this is the kind of cardiac disease which, more than any other, has been found coincident with the peculiar change in the kidney. Among 100 cases, recorded in a tabular form by Dr. Bright, there were 27 in which no affection of the heart could be detected. In 52 instances the heart presented the characters of hypertrophy, and of these no fewer than 34 were free from any trace of valvular disease. Among these 34 there were 11 cases of disease affecting the aorta: in the remaining 23 no cause for the existing hypertrophy and dilatation could be found in the heart itself, or in the great blood-vessels. The true cause may therefore be reasonably supposed to have been the renal disease, operating upon the involuntary muscle through the quality of the blood.

"Whether the renal disease be ever produced by the cardiac, is more questionable. In the acute renal affection, when it proves early fatal, the kidney is always found to be gorged with blood. And the customary intermixture of blood with the urine warrants the belief that the same condition was present in the patients who have recovered. From this state of engorgement springs, apparently, the subsequent series of changes. It is therefore a plausible conjecture that whatever tends to produce congestion of the kidney, tends, also, to aggravate, and may even cause, the peculiar changes in question. I need not now tell you that disease of the heart frequently occasions congestion of the venous system, and gorge the viscera with blood. Under this influence the liver often *enlarges.* On the other hand, disease of the heart, even such as gives rise to venous congestion and to dropsy, often lasts long, and proves ultimately fatal, without the occurrence of albuminous urine, and without any appreciable change of structure in the kidney.

"Pain or tenderness of the loins, is sometimes, and sometimes only, an accompaniment of the renal disease. This symptom is more often present in the early than in the later stages of the malady. It occurred in one-third of twenty-eight cases narrated by M. Solon. Dr. Gregory noticed it in the half of his patients.

"*Causes.*—The *causes* of the disease of which I have been endeavoring to sketch the outline, are often obscure. Its more obvious symptoms, in the chronic form of the malady, have been observed, in very many instances, to begin soon after the exposure of the body to wet and cold under unfavorable circumstances. But it is by no means certain—indeed the probabilties preponderate on the other side—that, in these instances, the renal disorder had not previously existed in a latent state.

"It is certain, however, that the acute kidney affection, which may be considered identical with febrile dropsy, does often arise under similar circumstances of exposure, and is attended with a marked disturbance of the functions of the kidneys. And Brights disease, in its chronic form, has been noticed as occurrmg in persons who had previously suffered, and had apparently recovered from, an attack of febrile dropsy. Are we not warranted in believing that the recovery was *imperfect* in such cases? that the kidney had sustained irretrievable injury? and that the disease, although from the treatment employed, or by lapse of time, it had become tranquil or latent, was ready again to give indications of its existence upon any repetition of its exciting cause?

"Again, it is matter of common observation that intemperate habits have often preceded the development of this disease. Yet we may conclude that intemperance in drinking is rather a predisposing than an essential cause, from the fact that the malady is not unknown among children, and other persons whose manner of life has been strictly sober. I had lately an example of this in a young girl, fifteen years old, who had never menstruated. And this leads me to remark that the renal disorder has been known, in many instances, to follow a sudden check or suppression of the catamenia. It has sometimes seemed to owe its origin to blows received upon the loins.

"The complaint happens at all ages: less often, however, in extreme youth than afterwards. Sabbatier records that he saw, while in the service of M. Baudelocque, a young infant affected with anasarca and albuminous urine. The first case described by M. Solon, is that of an infant, seventeen months old, in whom similar symptoms appeared shortly after exposure to cold and wet. In 1838 a boy, between five and six years old, anasarcous, and passing bloody and albuminous urine, was in the Middlesex Hospital, under the charge of my colleague Dr. Wilson. M. Constant, in the *Gazette Médicale*, for 1835, cites the case of a child of five years of age; and M. Rayer gives two plates, representing the kidneys of two children, the one five and the other six years old, who both died of dropsy with albuminous urine, the sequel of scarlet fever. In each of these the changes described by Dr. Bright were well marked, and the bulk of the kidney was considerably increased.

"The malady is, however, much more common in adults; not, in all probability, because the kidney is more readily susceptible of it at one period of life than another, but because, as life advances, the circumstances which tend to produce or foster it become of more frequent operation; intemperance, exposure to great vicissitudes of temperature, and (perhaps) disease of the heart.

"It occurs, I presume for the same reasons, oftener in men than in women.

"Dr. Christison suspects that Bright's kidney happens chiefly in persons of scrofulous habit; and he found it, in several instances, coincident with phthisis pulmonalis. My own experience would not have led me to that opinion. I partake in M. Solon's doubts, whether the coexistence of the pulmonary consumption and of this renal malady is more than casual. Dr. Bright tells us that 'the instances in which phthisis, or any form of scrofulous or tubercular disease, has been connected with the renal affection, have been decidedly rare.'

"*Nature of the Disease.*—What, after all, is the true character and essence of the organic metamorphosis which constitutes this formidable disorder, Bright's kidney? All that has been ascertained of its early stages, of its course, and of its causes, furnishes to my mind a strong presumption that the structural change, in all its varieties of aspect, may be ultimately traceable to an undue accumulation and stagnation of blood in the blood-vessels of the kidney. Those curious arterial bunches, the Malpighian bodies, appear especially to be overfilled and obstructed. Rayer calls the complaint *albuminous nephritis;* and perhaps the congestion (which unquestionably is present in what I consider the acute form of the malady) may sometimes pass into chronic inflammation. We do not, however, find that it ever terminates in suppuration; yet suppuration is no uncommon event of true inflammation of that part, excited by violent injuries, or by the lodgment of calculi within it. It seems to me more probable that the mischief done to the kidney is owing to extreme congestion, and its usual consequences—the oozing forth of the blood in substance, or of some of its constituents, into the interstitial textures, as well as into the excretory tubes of the kidney. The appearance of these ingredients of the blood, and even sometimes of blood itself, in the urine; the increased size of the gland in the earlier stages; the various shades of color which its surface and parts of its interior present, as the coloring matters of the effused fluids are more or less absorbed; the impermeability of those altered parts by artificial injections; the ultimate shrinking and hardness of the organ as the disorder becomes chronic, and absorption proceeds; these are all consistent with this theory. It is plain that the morbid conditions of the urine depend, in part at least, upon the mechanical transudation of certain portions of the blood, which pass through the kidney unchanged, as through an inert filter. Mixed with the urine we find serum, with its albumen, and its salts, which diminish the acidity of the mixture, or even render it neutral; and in many cases we find more or less of the coloring matter also of the blood. Those portions of the extravasated fluid which have no outlet of escape, solidify, and thus obliterate the natural texture of the part they have invaded. The obstruction of the emergent veins of the kidney by firm clots of blood is in harmony with the same supposition.

"When the kidney is thus spoiled, its natural function is imperfectly or but partially performed. The change which it should effect upon the blood, by purifying it from urea, fails to be accomplished. The albuminous impregnation, and the other altered qualities of the urine when voided, may be explained either by supposing that the secreting power of the whole gland is interfered with, but not absolutely suspended; so that the urine is incompletely elaborated; or, by supposing that portions of the gland are spoiled, and portions remain sound and effective; that true urine is formed by the healthier portions, and mixes with the constituents of the blood which pass, as such, through the diseased portions. The latter of these hypotheses is most in accordance with the fact that, in the advanced stage of the disorder (when we may conceive the spoiled parts to have become mere solid unchanging masses) the albumen is apt to disappear from the urine: and also with the fact, that complete recovery does sometimes appear to take place; in which cases we may imagine that, although a small portion of the substance of the gland has undergone irremediable change, enough of it remains healthy to serve the wants and purposes of the economy."

The style of these lectures, as the foregoing extracts will bear testimony, is concise, simple and perspicuous. So much information could hardly have been imparted in fewer words. No details necessary to the full elucidation of the subject are omitted, but at the same time that the reader experiences this to be so, he will admit that, in the minuteness of his descriptions, the author never grows tedious. We are free to state, that a careful examination of the volume has satisfied us, that it merits all the commendation which has been bestowed upon it at home, and in this country. It is a work adapted to the wants of the young practitioner, combining as it does sound principles and substantial practice. It is not too much to say, that it is a representative of the actual state of medicine as taught and practised by the most eminent physicians of the present day, and as such we would advise every one about embarking in the practice of physic to provide himself with a copy of it. Y.

